# Research on household emergency supplies storage from the theory of planned behavior and intention-behavior gap in the context of COVID-19

**DOI:** 10.3389/fpsyg.2022.1069843

**Published:** 2023-01-16

**Authors:** Luyan Wang, Yuan Jiang, Liujun Pan, Jiangyuan Ji, Aoao Xu

**Affiliations:** ^1^School of Finance and Economics, Jiangsu University, Zhenjiang, China; ^2^Macquarie Business School, Macquarie University, Macquarie Park, NSW, Australia

**Keywords:** public health emergencies, household emergency supplies storage, theory of planned behavior, intention-behavior gap, structural equation modeling

## Abstract

**Introduction:**

In the context of COVID-19 epidemic, household-level emergency supplies are becoming a critical link in the national emergency response mechanism for public health emergencies. The main goal of this study is to analyze the forming process of household emergency supplies storage intention and behavior based on the theory of planned behavior.

**Methods:**

A total of 486 valid questionnaires were obtained from China and analyzed using structural equation modeling.

**Results:**

The study found that subjective norms and perceived behavioral control had a positive impact on residents’ intention to store emergency supplies, while attitudes did not play a significant role. Community institutional trust and community network play significant moderating roles in the transformation from intentions to behaviors.

**Discussion:**

This study explored the influencing factors of residents’ household emergency supplies storage, and introduced community institutional trust and community network as moderating variables to analyze the process of transformation of residents’ household emergency supplies storage intentions to behaviors from the perspective of community situation, and initially constructed a two-stage integration model including intention formation and behavior transformation. By analyzing the forming process of household emergency supplies behavior, this paper revealed the effective paths for the formation of household emergency supplies storage intention, and put forward policy suggestions from the government and community levels.

## 1. Introduction

The outbreak and spread of COVID-19 in 2020 have caused enormous economic losses and public health pressure around the world (Yin et al., 2022), and public health emergencies have attracted the attention of many scholars. In the context of environmental uncertainty and ecosystem instability (Wang et al., 2020), the threat of traditional infectious diseases persists and new types of infectious diseases are constantly emerging. The frequency of public health emergencies has been increasing, which is difficult to predict, harmful and widely spread. It not only seriously affects the living standard and quality of residents, but also threatens their lives and health. Therefore, residents and households, as the most likely victims of public health emergencies, should establish a certain awareness of household emergency supplies storage to enhance the household’s ability to respond to public health emergencies. At the same time, as the smallest unit of society, the household is an important subject that cannot be ignored in the construction of resilient cities (Chu et al., 2021; Hananel et al., 2022) in the context of public health emergencies, and is a key link in the construction of a multi-level, systematic and all-round public health emergency response system. Household emergency supplies storage has become an important indicator of a country’s emergency materials comprehensive support capacity and level. Household emergency supplies storage in the context of public health emergencies refers to the routine storage of some emergency supplies (including food, drinking water, masks and disinfectant solution) by households to cope with the possible adverse effects of future public health emergencies (Paton, 2019; Dasgupta et al., 2020). According to the survey, Chinese residents’ awareness of household emergency supplies storage is not high, so the study of household emergency supplies storage based on the context of COVID-19 has strong theoretical and practical significance.

Actions speak louder than words, and it is more important to actually store household emergency supplies than to establish a high intention to store in terms of improving the households’ ability to respond to public health emergencies. However, in a study of typhoon disaster preparedness, Ng (2022) noted that although there was a significant correlation between residents’ intentions and behaviors in terms of disaster preparedness, the level of behavior was significantly lower than that of intention. During our team’s preliminary research, this interesting phenomenon was also noticed, that is, even though urban residents have a high intention to store household emergency supplies, fewer of them actually turn into storing behaviors, and there may be an intention-behavior gap in the field of household emergency supplies storage. Therefore, the transformation process of storing intentions to behavior should receive special attention and research, and it is necessary to investigate which factors affect the transformation of urban households’ intentions to actual storing behavior.

Nowadays, a large number of studies have focused on panic buying behavior in the context of COVID-19 (Fu et al., 2021; Kassas and Nayga, 2021) and preparedness for different types of disasters (e.g., typhoons, earthquakes, etc.; Zaremohzzabieh et al., 2021; Hu et al., 2022; Ng, 2022). However, there are relatively few studies on the behavior of household emergency supplies storage. As an important component of disaster preparedness, household emergency supplies storage deserves more focus under the background of normalization of epidemic prevention and control. And research is also needed to provide theoretical support for the intention-behavior gap in the field of disaster preparedness. Based on the theory of planned behavior, this study explored the influencing factors of intention to store household emergency supplies in the context of COVID-19, and also examined the moderating effects of community institutional trust and community network on the transformation of intention into behavior of household emergency supplies storage. And the marginal contributions may include: (1) Switching the research perspective to focus on household emergency supplies storage, and explore the influencing factors of urban household emergency supplies storage under the background of normalization of epidemic prevention and control. (2) Focusing on the intention-behavior gap, analyzing the transformation process of intention into behavior of urban household emergency supplies storage, and empirically demonstrating the moderating role of external contextualized factors such as community institutional trust and community network in the transformation process.

The rest of this paper is organized as follows. Section 2 reviews the literature on the theory of planned behavior, disaster preparedness, community institutional trust, and community network and presents the research hypotheses. Section 3 describes the questionnaire used in the study, the process of data collection, and the demographic characteristics of the respondents. Section 4 summarizes the evaluation results of the measurement and structural models in this study. Section 5 discusses the conclusions and policy implications derived from this study. Section 6 discusses the shortcomings of this study and future research directions.

## 2. Literature review and research hypotheses

### 2.1. Theory of planned behavior

**Figure 1:**
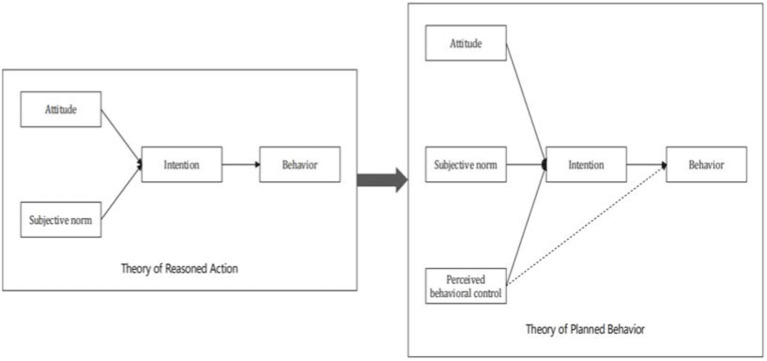
From TRA model to TPB model.

The theory of planned behavior (TPB) is based on the theory of reasoned action (TRA; Ajzen and Fishbein, 1973), which holds that both attitudes (the positive or negative feelings an individual has about his/her behavior) and subjective norms (the social pressure an individual feels to perform a particular behavior) simultaneously influence intentions and behavior is directly driven by intentions. However, it is found that TRA has some defects in behavior prediction, because most behaviors in real life are not only influenced by individuals’ subjective intention, but also affected by external conditions and self-ability. Therefore, Ajzen (1991) proposed the TPB model (shown in Figure 1) by adding the variable of perceived behavioral control (how easy it is for an individual to accomplish certain specific behaviors) to the theory of reasoned action. Armitage and Conner (2001) argued that TRA and TPB differed in their applicability, with TRA being better at predicting relatively simple behaviors and TPB being more comprehensive and accurate when predicted behaviors are subject to certain constraints. Household emergency supplies storage is not only closely related to the individuals’ attitudes and the social pressure they feel, but also affected by time, income, storage skills and other factors. Therefore, this study concluded that TPB was more suitable than TRA in terms of household emergency supplies storage.

#### 2.1.1. Attitude

Attitudes are the positive or negative views that individuals hold towards particular behaviors (Ajzen, 1991) and can be primarily categorized as cognitive, emotional, and behavioral (Eagly and Chaiken, 2007). When individuals have positive attitudes towards storing household emergency supplies and believe that doing so will significantly improve their ability to respond to public health emergencies both individually and as a family, they are more likely to store household emergency supplies. In the TPB model proposed by Zaremohzzabieh et al. (2021), the attitudes have a significant positive effect on household disaster preparedness, therefore, hypothesis 1 is proposed in this paper.

*H1*: Attitude positively affects household emergency supplies storage intention.

#### 2.1.2. Subjective norm

Subjective norms refer to the social pressure individuals feel when they perform a particular behavior (Ajzen, 1991). When important people around them believe that a certain behavior should be done, individuals feel pressured by normative beliefs and the motivation to comply, and tend to meet the expectations of others and society. Numerous studies (e.g., Najafi et al., 2017; Tan et al., 2020; Ng, 2022) have shown that subjective norms are important factors in influencing disaster preparedness. In the model constructed by Ng (2022), the subjective norm is the only variable among the three basic prior variables of TPB (attitude, subjective norm, and perceived behavioral control) that has a significant effect on preparedness intentions. The intention to store household emergency supplies will improve when individuals are aware that family members, neighbors, friends, etc. hold that they should do so in the context of COVID-19, so hypothesis 2 is proposed in this paper.

*H2*: Subjective norm positively affects household emergency supplies storage intention.

#### 2.1.3. Perceived behavioral control

Perceived behavioral control refers to an individual’s perception of his/her ability to engage in a certain behavior (Ajzen, 1991). When attitudes and subjective norms reach a certain level, non-intention factors perceived by the individual, such as opportunities and resources, can play an important role in behavior (Ajzen, 1991), i.e., when an individual has a positive attitude toward a behavior and important people around him also agree that a behavior should be taken, and when the individual has greater control over certain external factors that are outside his/her complete control, the greater the intention of an individual to perform a particular behavior, the more likely the individual will perform that particular behavior. In the case of household emergency supplies storage, individuals will think about whether they have the ability (including time, money and skills) to store, and when individuals think they have less ability or perceive more uncontrollable factors, their intention to store household emergency supplies will decrease, and they will tend not to actually store household emergency supplies, so hypothesis 3 and 4 are proposed in this paper.

*H3*: Perceived behavioral control positively affects household emergency supplies storage intention.

*H4*: Perceived behavioral control positively affects household emergency supplies storage behavior.

#### 2.1.4. Intention

Although there are fundamental conceptual differences between an individual’s intention and behavior, human behavior in general is a concrete action expression of intention (Ajzen, 1991). The higher the intention to perform a behavior, the greater the likelihood that the behavior will actually occur. Current studies (e.g., Zaremohzzabieh et al., 2021; Ng, 2022) suggest that intention has a positive effect on disaster preparedness behavior, and that higher intention to store household emergency supplies is more likely to lead to storage behavior. Therefore, hypothesis 5 is proposed in this paper.

*H5*: Storage intention has a positive effect on household emergency supplies storage behavior.

### 2.2. Moderating effects of community institutional trust and community network

Although intention is a crucial reference point and foothold for predicting individuals’ actual behavior, there is a large gap between their intention and their subsequent behavior (Bagozzi, 1992), that is, the intention-behavior gap. The theory of social cognition suggests that individuals, behaviors, and contexts form a complex system, and that external contextual factors can influence the process of environmental stimulation on individuals’ cognition, which in turn leads to changes in subsequent behavior (Bandura, 2002). External contextual factors are the objective environment that individuals face when performing a particular behavior and give rise to the intention-behavior gap. The intention-behavior gap has been demonstrated in many areas such as sustainable consumption (Diddi et al., 2019; Sultan et al., 2020; Rausch and Kopplin, 2021), pro-environmental behavior (Echegaray and Hansstein, 2017; Mack et al., 2019; Hua and Dong, 2022), and energy (Fang et al., 2021; He et al., 2022). Relevant research shows that contextual factors can promote or hinder the implementation of preparedness decisions (Martins et al., 2019), and not all individuals will implement their behavioral intentions (Ng, 2022). Combined with the model of “Attitude-Behavior-Context,” this study believes that the external environmental factors faced by urban residents-community should be deeply analyzed. The community is an important social unit that influences individuals’ preparedness behavior (Kirschenbaum, 2004) and has become a fundamental research unit for assessing disaster preparedness behavior. The intention of individuals to actually store household emergency supplies is closely related to external contextual factors such as community environment and community strength. Community can be interpreted from the perspectives of geographical space and government administration (Liang and Zhong, 2022). Community is not only a spatial unit composed of a large number of residential complexes, but also one of the important participants in grassroots governance. Most of the existing literature studies the internal structure of communities in terms of these two dimensions (e.g., Kirschenbaum, 2004; Sharp et al., 2013; Zaremohzzabieh et al., 2021; Gamo and Park, 2022). Therefore, this study considers that the intention-behavior gap in household emergency storage can be explored from a community perspective at two levels: first, community institutional trust based on governance capacity and service levels; and second, community networks based on interactive connections, human relationships, and shared visions.

#### 2.2.1. Community institutional trust

Community institutional trust refers to community residents’ confidence in the ability of community institutions to make effective decisions based on full consideration of residents’ interests (Houston and Harding, 2013). Trust can be divided into institutional and non-institutional trust, with institutional trust built on established operational systems and non-institutional trust mainly consisting of interpersonal trust. This study argues that if the research perspective is focused on the community context, institutional trust and non-institutional trust are like the community institutional trust and community network studied in this section, respectively. According to Blomqvist (1997) and Luhmann (1988) institutional trust precedes interpersonal trust, and community institutional trust has a greater impact on individual behavioral decisions than community networks. In the context of COVID-19, the higher the level of trust established based on the past performance of community institution, the more community residents trust the disaster information released by it and are more willing to actually store household emergency supplies to cooperate with the community’s epidemic prevention and control, which plays a positive moderating role in the transformation of intention to behavior, thus hypothesis 6 is put forward in this paper.

*H6*: Community institutional trust plays a positive moderating role in the transformation of the intention to store into storing behavior.

#### 2.2.2. Community network

Community network refers to the relatively stable relationship system formed by interaction among community residents (Wasserman and Faust, 1994), which provides an ecosystem of mutual influence among residents (Zaremohzzabieh et al., 2021). As an essential part of social networks, community networks are also characterized by safety (a sense of trust and cohesion, social support that is exchanged equally) and effectiveness (the existence of distinct motives, the pursuit of efficacy, profit, and control) of social networks (Kadushin, 2002). However, when it comes to public health emergencies such as COVID-19, individuals tend to regard ‘safety’ and ‘trust’ as their basic motives for survival and self-protection, and the safety features of community networks will be enhanced and enlarged, which then is transformed into the unique behaviors in community networks, such as the communication of information about the epidemic and exchange of status of household emergency supplies. As community network based on physical boundaries gathers relatively homogeneous and tight-knit groups, individuals are more likely to feel the necessity of actual storing when other individuals in the network store household emergency supplies in case of emergency. As a result, community network plays a positive role in moderating the transformation from intention to behavior, thus this paper proposes hypothesis 7.

*H7*: Community network plays a positive role in moderating the transformation from the intention to store into storing behavior.

**Figure 2:**
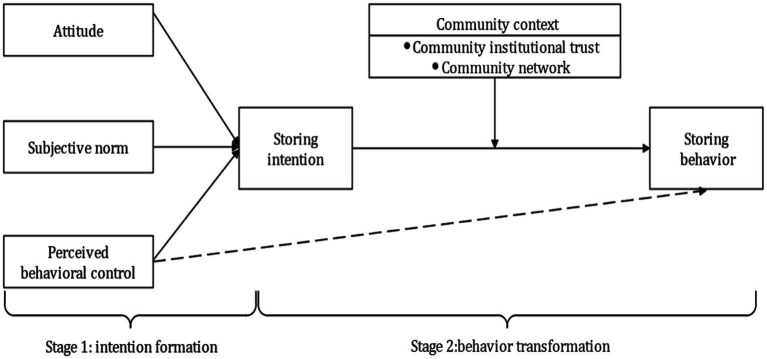
Research model.

In summary, based on the theory of planned behavior and the intention-behavior gap, this paper constructs a two-stage integration model that includes intention formation and behavior transformation around household emergency supplies storage in the context of COVID-19 (as shown in Figure 2). The first stage of the model focuses on the generation of the intention to store household emergency supplies. It is based on the theory of planned behavior and aims to clarify the specific paths and mechanisms of attitudes, subjective norms, and perceived behavioral control on the intention to store household emergency supplies. The second stage of the model focuses on the transformation from intention to behavior, and aims to explore the influence of external contextual factors, such as community institutional trust and community network, in the transformation from intention to behavior.

## 3. Research methodology

### 3.1. Questionnaire

The questionnaire in this study was divided into three main sections: (1) the research background and the concept of household emergency supplies storage; (2) demographic variables such as gender, age, education, monthly family income and family size; (3) measure items of attitude (ATT), subjective norm (SN), perceived behavioral control (PBC), storing intention (SI), storing behavior (SB), community institutional trust (CIT), and community network (CN). All scales were adapted from Ajzen (1991), Grimmelikhuijsen (2012), Kohn et al. (2012), Najafi et al. (2017), Daellenbach et al. (2018), Ru et al. (2018), Martins et al. (2019), Tan et al. (2020), Mansoor (2021), and Ng (2022), etc. (shown in Appendix A). All items were measured with a five-point Likert scale, where 1–5 indicate very disapproval and very approval, respectively.

In order to improve the reliability and accuracy of the questionnaire measurement, this study improved the questionnaire from the following three aspects. First, three professors and five graduate students were invited to form an expert group to evaluate the questionnaire and revise the content and language of the questionnaire. Second, the interviewees were asked about their feelings and problems in answering the questions, and according to their feedback and suggestions, the expression of the questionnaire was improved. Finally, according to the results of the pre-survey, the measure items whose reliability did not meet the standard were adjusted.

### 3.2. Procedure and sample

This research chooses urban households in Jiangsu Province as the research sample mainly based on the following considerations. First, in the context of population growth, increased life expectancy and the level of urbanization, cities and towns with greater population density, more frequent population movements, and more active human activities are considered to be the most vulnerable areas to potential disasters (Ribeiro et al., 2022). The damage and consequences of public health emergencies such as COVID-19 are frequently worse in towns than in non-urban areas such as rural areas, so it is particularly crucial for urban residents to be aware of the need to store household emergency supplies. The urbanization rate of Jiangsu Province in China is currently at a high level throughout the country. According to statistics, Jiangsu Province’s urbanization rate reached 73.44% in 2020, which was nearly 10% higher than the national average. In view of this, this paper focuses on Jiangsu Province, China, and urban residents in Jiangsu Province are selected as the research subject. Second, this paper mainly explores the urban residents’ intention to store household emergency supplies under the background of the normalization of epidemic prevention and control, which requires control for the phased stress response caused by the recent external disaster factors and requires the relative stability of the living environment in the sample area. Jiangsu Province in China has not been threatened by a large-scale disaster in the past 6 months. It has a good living environment and its residents live stable lives, but it is also frequently affected by COVID-19 on a small scale and has a certain risk perception ability, therefore, this paper selects urban residents in Jiangsu Province of China as the research sample.

The quantitative data used in this study were collected through an offline household survey, and the data were collected from August to October 2022. First, four prefecture-level cities in Jiangsu province were systematically selected as the study sample. Second, four counties (districts) were selected in each of the four prefecture-level cities. Again, four urban communities were systematically selected from the sampled counties (districts). Finally, eight households in each urban community were selected by simple random sampling method, and adult residents above 18 years old were invited as interviewees. To improve the reliability of the data and the correctness of the estimation, each researcher was systematically trained and required to explain the background of the research and the confidentiality of the data to the interviewees before the research was started. For some dyslexic interviewees, the team arranged research assistants to read out the questions in a way they could understand without affecting their answers. In the end, the team collected a total of 510 questionnaires and eliminated 24 invalid questionnaires with obvious logical errors and the same answers for most projects, resulting in 486 valid questionnaires.

The demographic characteristics of the respondents are summarized in Table 1. The number of male (49.8%) and female (50.2%) respondents was almost equal. The majority of respondents (73.2%) were in the middle-aged and young adult group between the age of 26–55, with relatively few older adults (7.1%) aged 56 and older. A vast number of respondents (83.1%) had a college degree or higher, and respondents as a whole had a high level of education. In terms of household income, more than two-thirds of the respondents (69.7%) showed that their monthly household income was between RMB 8,001 and RMB 20,000, 15.0% had a monthly household income of less than RMB 8,000, and 15.4% had a monthly household income of more than RMB 20,001. In addition, more than 60% of respondents (63.9%) have 3–5 family members living together permanently in their households.

**Table 1:** Respondent profile (*N* = 486).

Characteristics	Options	Frequency	Percent (%)
Gender	Male	233	49.8
	Female	235	50.2
Age	Under 25	92	19.7
	26–35	120	25.6
	36–45	139	29.7
	46–55	84	17.9
	56 or above	33	7.1
Education	Junior middle school or below	27	5.8
	Senior high school (vocational school)	52	11.1
	Junior college	178	38
	Bachelor degree	172	36.8
	Master’s degree or PhD	39	8.3
Monthly family income (RMB)	Less than 8,000	70	15
	8,001–12,000	122	26.1
	12,001–16,000	123	26.3
	16,001–20,000	81	17.3
	20,001 or above	72	15.4
Family size	1	24	5.1
	2	58	12.4
	3	132	28.2
	4–5	167	35.7
	6 or above	87	18.6

## 4. Data analysis and results

In this study, Mplus8.0 was used to construct the measurement model (Appendix B) and the structural model (Appendix C; Anderson and Gerbing, 1988) for analysis. The measurement model is mainly used to test the relationship between structures and corresponding items, while the structural model is mainly used to measure the relationship between structures (Ru et al., 2018). In this part, the reliability, convergent validity and discriminant validity of the measured items are obtained by confirmatory factor analysis (CFA), the relationship between the measured items and the reliability of the structure are evaluated, and then the structural equation model is constructed to test the above research hypotheses.

This study used confirmatory factor analysis (CFA) to estimate the reliability and validity of the model. As shown in Table 2, the minimum value of Cronbach’ α is 0.836, the minimum value of composite reliability is 0.836, which is greater than the recommended minimum value of 0.700, and the KMO value is greater than 0.700. Bartlett’s spherical test is significant. The standardized load coefficient of the measurement items of the scale is between 0.765 and 0.887, and the average extraction variance is above 0.5, indicating that the measurement model has good convergence validity. As shown in Table 3, the AVE square root of all potential variables in this study is greater than the absolute value of the correlation coefficient between this variable and other variables, indicating that the measurement model has good discrimination validity. Confirmatory factor analysis (CFA) showed that the scale had good construct validity.

**Table 2:** Confirmatory factor analysis.

Construct	Indicator	Standardized loading	Cronbach’s alpha	Composite reliability	AVE
Attitude	ATT01	0.811***	0.886	0.887	0.724
	ATT02	0.852***			
	ATT03	0.887***			
Subjective norm	SN01	0.847***	0.853	0.855	0.662
	SN02	0.811***			
	SN03	0.782***			
Perceived behavioral control	PBC01	0.835***	0.839	0.841	0.638
	PBC02	0.765***			
	PBC03	0.794***			
Storing intention	SI01	0.875***	0.88	0.881	0.712
	SI02	0.874***			
	SI03	0.779***			
Storing behavior	SB01	0.849***	0.883	0.884	0.717
	SB02	0.854***			
	SB03	0.837***			
Community institutional trust	CIT01	0.775***	0.836	0.836	0.63
	CIT02	0.799***			
	CIT03	0.807***			
Community network	CN01	0.860***	0.879	0.88	0.709
	CN02	0.812***			
	CN03	0.853***			

Significant at ***p < 0.001.

**Table 3:** Descriptive statistics and correlations.

Construct	Convergence validity								
	Means	SD	1	2	3	4	5	6	7
ATT	3.283	1.019	**0.851**						
SN	3.311	1.135	0.474	**0.814**					
PBC	3.192	1.058	0.489	0.539	**0.799**				
SI	3.548	1.122	0.419	0.621	0.602	**0.844**			
SB	2.74	1.068	0.318	0.3	0.41	0.381	**0.847**		
CIT	2.702	0.799	0.028	0.117	0.032	0.241	−0.447	**0.794**	
CN	3.28	1.177	0.16	0.18	0.16	0.161	0.602	−0.26	**0.842**

Means are measured based on average factor scores; SD means standard deviation; values in the diagonal row (bold) are the square roots of AVEs and the others are the correlations between constructs.

### 4.1. Structural equation modeling

In this study, structural equation model is used to explore the relationship between the results. Model 1 is the original TPB model; in model 2, the moderating variable of community institutional trust is added in the process of transformation from intention to behavior; in model 3, the moderating variable of community network is added in the process of transformation from intention to behavior. The fitting index of model 1 is Chi-squared/df = 1.267, RMSEA = 0.024, CFI = 0.995, TLI = 0.993, SRMR = 0.032; the fitting index of model 2 is Chi-squared/df = 1.416, RMSEA = 0.030, CFI = 0.986, TLI = 0.983, SRMR = 0.043; the fitting index of model 3 is Chi-squared/df = 1.316, RMSEA = 0.026, CFI = 0.990, TLI = 0.988, SRMR = 0.031. The model is well fitted and acceptable.

As shown in Table 4, model 1 explains 48.7% of the variance of the intention to store, but only 20.2% of the variance of storage behavior. This not only shows that TPB model has a good explanatory power for the intention to store household emergency supplies, but also proves that there is a gap between the intention and behavior. It is necessary to introduce moderating variables for further analysis. In Model 2 and Model 3, two moderating variables, community institutional trust and community network, are introduced based on TPB model. The explanatory power of these two variables to storage behavior increased to 50.4% and 51.8%, respectively, which is significantly improved compared with model 1.

**Table 4:** Hypotheses testing results.

Hypothesis (relation)	Model 1			Model 2			Model 3		
	Standardized path coefficient	Standard error	Decision	Standardized path coefficient	Standard error	Decision	Standardized path coefficient	Standard error	Decision
H1 (ATT → SI)	0.05	0.052	Rejected	0.049	0.051	Rejected	0.05	0.051	Rejected
H2 (SN → SI)	0.404***	0.052	Accepted	0.412***	0.052	Accepted	0.402***	0.052	Accepted
H3 (PBC → SI)	0.359***	0.055	Accepted	0.358***	0.054	Accepted	0.362***	0.054	Accepted
H4 (PBC → SB)	0.295***	0.064	Accepted	0.192***	0.058	Accepted	0.244***	0.055	Accepted
H5 (SI → SB)	0.206***	0.064	Accepted	0.327***	0.054	Accepted	0.171**	0.054	Accepted
H6 (CIT*SI → SB)				−0.142*	0.06	Rejected			
H7 (CN*SI → SB)							0.193***	0.043	Accepted
*R*^2^ (SI)	0.487			0.496			0.488		
*R*^2^ (SB)	0.202			0.504			0.518		

*p < 0.05, **p < 0.01, ***p < 0.001.

**Figure 3:**
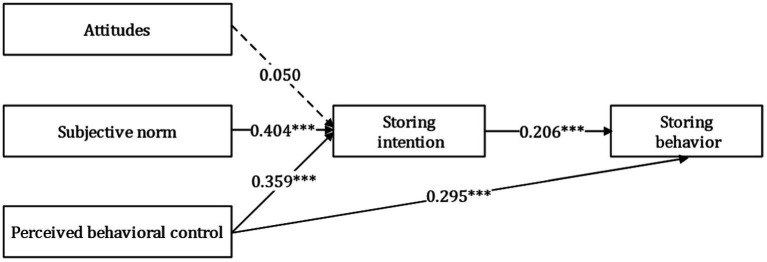
Diagram of model 1 path coefficient.

According to Table 4 and Figure 3, the standardized path coefficient from the attitude to the intention of households to store emergency supplies is 0.050, which is not significant at the 5% level. The two do not have statistical significance, so H1 has not been supported. This may be because attitudes cannot well explain intention and behavior in extreme situations such as public health emergencies (Turaga et al., 2010). At the same time, Glasman and Albarracín (2006) believed that stability is one of the important factors affecting attitude. However, in the context of the COVID-19 rising and falling, residents are disturbed by ambivalence such as anticipated regret (Loomes and Sugden, 1982) and experienced regret (Zeelenberg, 1996), so it is difficult to have a stable attitude towards the household emergency supplies storage. Therefore, in the context of COVID-19 the impact of attitudes on residents’ intentions are weak. The standardized path coefficient from subjective norm to storing intention is 0.404, which is significant at the 0.1% level and H2 is supported. The standardized path coefficient of this relationship is the highest, indicating that when family, friends, colleagues and other important influential people have high expectations for residents to store household emergency supplies, residents tend to show high storing intention due to group pressure, conformity psychology and other factors. The standardized path coefficient from perceived behavioral control to the intention of households to store emergency supplies is 0.359, and the standardized path coefficient from perceived behavioral control to the behavior is 0.295. Both are significant at the level of 0.1%, and H3 and H4 are supported. In many previous literatures, this path was invalid (e.g., Najafi et al., 2017; Tan et al., 2020), this may be because this study focuses on the normalized household emergency supplies storage in the context of COVID-19, which is somewhat different from the stressful household emergency supplies storage studied in previous literatures. Residents will fully consider whether they have the ability to carry out the normalized emergency supplies storage, so the perceived behavioral control has a significant impact on the storing intention and behavior. The standardized path coefficient from storing intention to storing behavior is 0.206, which is significant at the 0.1% level, and H5 is supported. It is worth noting that although this path is effective, the standardized path coefficient is only 0.206, indicating that the transformation rate between storing intention and storing behavior is not high, and the transformation from intention to behavior may be moderated by external situational factors.

**Figure 4:**
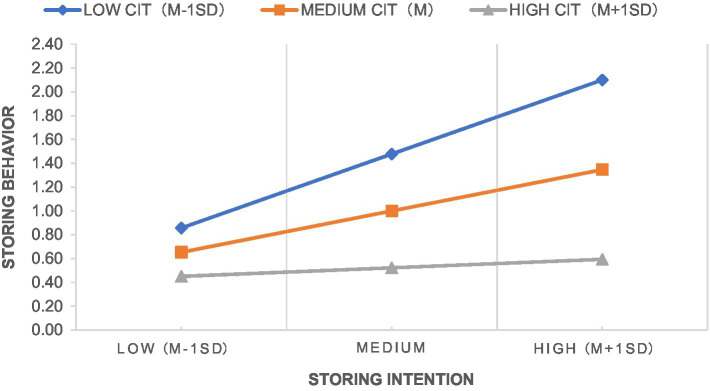
Diagram of the moderating effect of community institutional trust.

**Figure 5:**
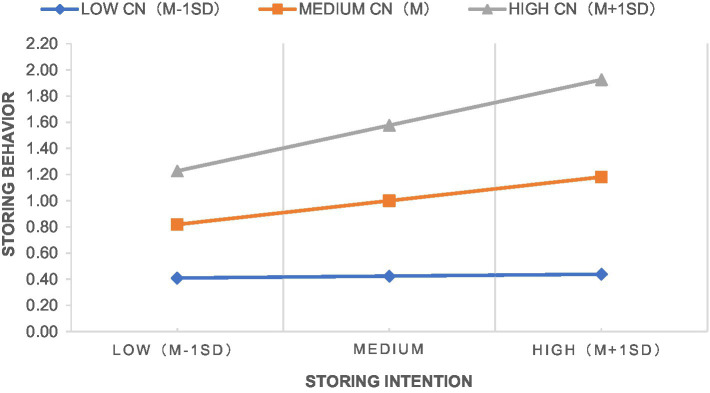
Diagram of the moderating effect of community network.

The interaction term of community institutional trust and storing intention was significant at 5% level, but the standardized path coefficient was negative, indicating that community institutional trust played a negative role in moderating the transformation from the intention to store household emergency supplies to storing behavior, which was contrary to H6. After experiencing the COVID-19, residents will evaluate the ability of their community to respond to public health emergencies, the stronger the ability of community institutions as grass-roots organizations, the higher the level of residents’ trust, as well as the belief that community institutions will take strong measures to ensure residents’ health and daily life and that household-level storage of emergency supplies is not necessary. At the same time, a large number of literatures believe that trust can be divided into two dimensions: trust and trustworthiness (Mayer et al., 1995; Rousseau et al., 1998). As a grassroots organization with close ties to the local government, the trust of residents may be transformed into a psychology of dependence, which in turn is manifested as not actually storing household emergency supplies. Therefore, community institutional trust plays a negative role in moderating the transformation from the storing intention of households’ emergency supplies into behavior. The interaction term of community network and storing intention was significant at 0.1% level, indicating that community network played a positive role in moderating the transformation from the intention to store household emergency supplies to storing behavior, and H7 is supported. Community network can help individuals expand the range and scope of obtaining and integrating information resources, and strengthen the exchange and communication among community members. Bansal and Voyer (2000) showed that communication intensity had a positive impact on individual behavior. Individuals are more likely to feel the necessity of actual storing when other individuals in the network store household emergency supplies in case of emergency. Therefore, community network plays a positive role in moderating the transformation from the storing intention of households’ emergency supplies into behavior. Figures 4, 5 show the slope analysis of the moderating effects of community institutional trust and community network, respectively.

## 5. Conclusion and implication

This study explores the influencing factors of urban household emergency supplies storage under the background of normalization of epidemic prevention and control, and analyzes the transformation process of intention into behavior of urban household emergency supplies storage from the perspective of willing-behavior gap. Based on the TPB model, the study introduced two external moderating variables: community institutional trust and community network, and constructed a two-stage integration model of behavior to store household emergency supplies including intention formation and behavior transformation. Using the research data of urban households in Jiangsu Province of China, the study draws the following conclusions:

(1) There is a gap between intention to store household emergency supplies and storing behavior, and the transformation rate from intention to storing behavior is low. The results showed that the level of intention to store household emergency supplies (mean = 3.548 ± 1.122) was much higher than that of storing behavior (mean = 2.740 ± 1.068), and at the same time, intention explained only 20.2% of the behavioral variance in TPB, indicating that even if many residents had high intention to store household emergency supplies, it might be disturbed by various internal and external factors that would not actually generate storing behavior, i.e., there is a gap between intention and behavior.

(2) Subjective norms and perceived behavioral control are important factors in influencing household emergency supplies storage. The results showed that both subjective norms and perceived behavioral control had a significant positive effect on the intention to store household emergency supplies. Many previous studies have concluded that the effect of perceived behavioral control on preparedness intentions is not significant (Najafi et al., 2017; Ng, 2022), and the different conclusion in this study might be due to the fact that this paper focused on normalized household emergency supplies storage in the context of COVID-19, which was fundamentally different from the stressful preparedness activities studied by previous scholars. It is a cyclical process to normalize the household emergency supplies storage, so whether the residents have the ability to store has a significant impact on the intention.

(3) Community institutional trust and community network play a role in moderating the transformation from the intention to store household emergency supplies to storing behavior. The findings showed that higher community network could promote the transformation from storing intentions to storing behavior, while higher community institutional trust hindered the transformation. Previous studies have generally concluded that community institutional trust positively influences preparedness intention and behavior, which may be due to the fact that community institutional trust in the context of COVID-19 is more focused on the level of community crisis management, and when residents realize that the community has a strong sense of responsibility and better ability, it is easier for them to generate the psychology of dependence but not actually store the household emergency supplies.

Based on the above conclusions, this paper puts forward relevant suggestions from both the government and the community levels:

(1) The government should strengthen the publicity, education, and training of household emergency supplies storage, and enhance the ability and level of preventing and dealing with the public health emergencies at the micro level. Since subjective norms and perceived behavioral control are important factors in influencing the intention to store household emergency supplies, strengthening awareness-raising and organizing skills training will be beneficial to strengthen residents’ recognition of the different roles of government, enterprise and family in public health emergencies, and to enhance self-protection consciousness and social responsibility.

(2) The community should continuously improve its own capacity and level of public health emergency management, and strive to create a harmonious and mutually supportive community atmosphere. Some residents are dependent on community institutions, so community organizations should actively take responsibility to improve their emergency response capabilities. Since community network plays a positive role in moderating the transformation from the intention to store household emergency supplies into behavior, community organizations should guide residents to build a community network of solidarity, civilization, harmony and mutual trust in the course of community activities.

## 6. Limitation and recommendation

Although this study provides a relatively complete study on household emergency supplies storage in the context of COVID-19, there are still inevitable deficiencies. First, the data in this paper mainly rely on self-report. In order to cater to the current mainstream values, residents may overestimate their intention to store. Second, this paper is based on the classical theory of planned behavior, and the model is relatively simple. Subsequent study will incorporate variables such as social network, disaster experience, government trust, and regret psychology to construct a model with more explanatory and predictive power. Third, this study only considers emergency storage at the household level, but in fact emergency storage is a multi-level and systematic project involving government, enterprises and households, and it will be more valuable and meaningful to include all the three in the model.

## Data availability statement

The raw data supporting the conclusions of this article will be made available by the authors, without undue reservation.

## Ethics statement

The studies involving human participants was conducted in accordance with the Declaration of Helsinki. Written informed consent for participation was not required for this study in accordance with the national legislation and the institutional requirements.

## Author contributions

LW: writing-original draft, writing-review and editing, methodology and investigation. YJ: conceptualization and project administration. LP: software and data curation. JJ: data curation and investigation. AX: data curation. All authors contributed to the article and approved the submitted version.

## Conflict of interest

The authors declare that the research was conducted in the absence of any commercial or financial relationships that could be construed as a potential conflict of interest.

## Publisher’s note

All claims expressed in this article are solely those of the authors and do not necessarily represent those of their affiliated organizations, or those of the publisher, the editors and the reviewers. Any product that may be evaluated in this article, or claim that may be made by its manufacturer, is not guaranteed or endorsed by the publisher.
