# Supplementary material for: Research on household emergency supplies storage from the theory of planned behavior and intention-behavior gap in the context of COVID-19
Source: Front Psychol. 2023 Jan 16;13:1069843. doi: 10.3389/fpsyg.2022.1069843 (PMC9885126; doi:10.3389/fpsyg.2022.1069843)
Supplement: Supplementary file 1 [file Data_Sheet_1.pdf]

Appendix A. : Constructs and measurement items.

Constructs	Measurement items	References
ATT	I support storing household emergency supplies.<br>I think it is a wise action to store household emergency supplies.<br>I think storing household emergency supplies is useful to protect myself and my family.	Ajzen(1991);<br>Najafi et al.(2017);<br>Daellenbach et al.(2018);<br>Tan et al.(2020);<br>Ru et al. (2018);<br>Ng(2022);<br>Kohn et al.(2012)
SN	My family and friends think that I should store household emergency supplies.<br>In regard to storing household emergency supplies, doing what people think I should do is important.<br>I feel under social pressure to store household emergency supplies.	
PBC	I am capable of storing household emergency supplies in my daily life if I wanted to.<br>Storing household emergency supplies is an easy thing for me.<br>I have the knowledge and skills to store and rotate household emergency supplies.	
SI	I expect to store household emergency supplies.<br>I plan to store household emergency supplies.<br>I will store household emergency supplies.	

SB	I will make a list of the emergency supplies that my family needs to store.<br>I will store household emergency supplies in case of public health emergencies in my daily life.<br>I will regularly check and replace household emergency supplies that are approaching shelf life.	
CIT	The community institution is acting in the interest of the public.<br>The community institution is capable.<br>The community institution can carry out their duties effectively.	Grimmelikhuijsen(2012);<br>Mansoor(2021)
CN	People in my neighborhood know each other.<br>People in my neighborhood trust each other.<br>People in my neighborhood try to make it a safer place to live.	Martins et al.(2019)

Appendix B. : Measurement model based on Mplus8.0

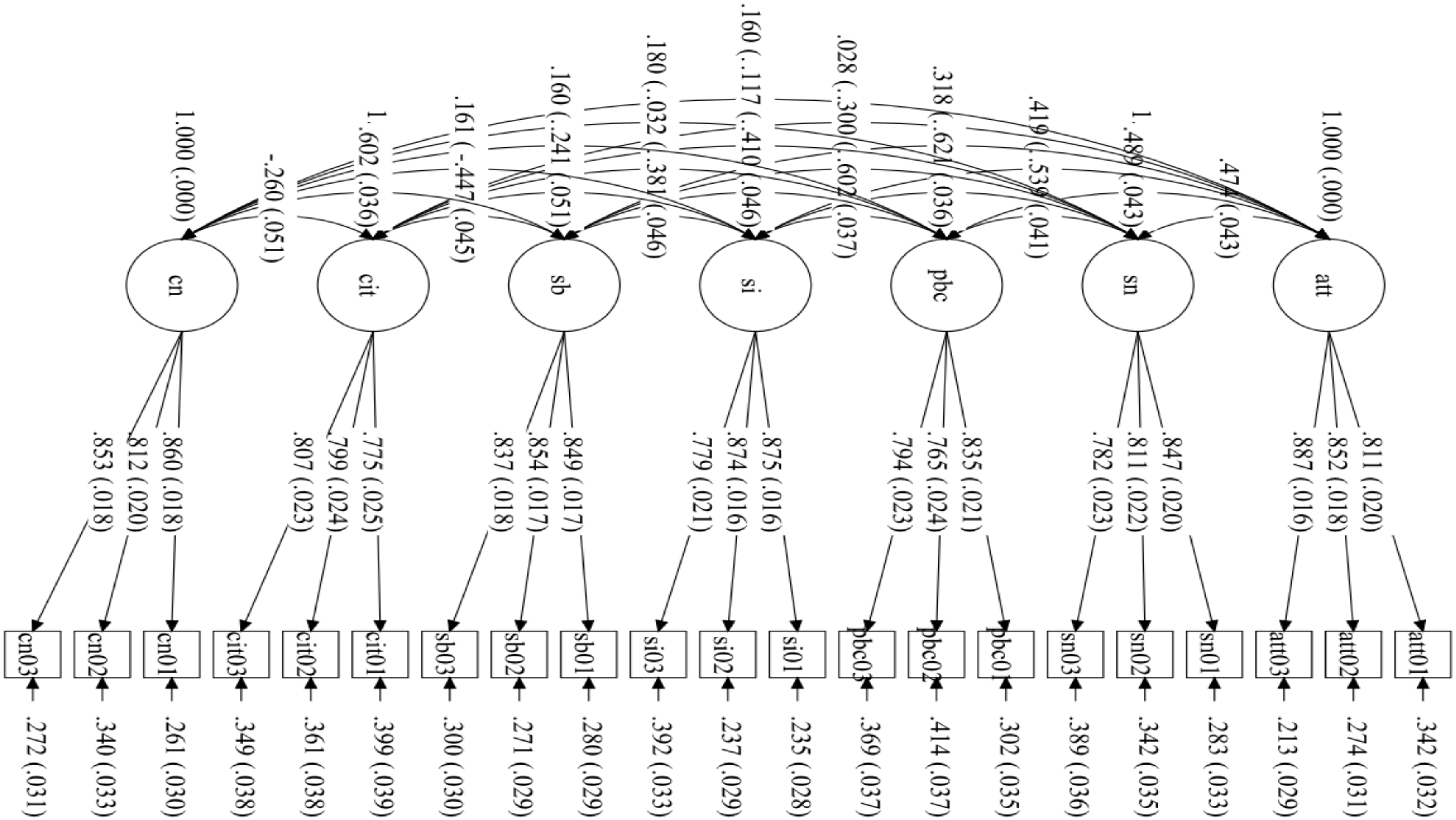

Appendix C. : Structural model based on Mplus8.0

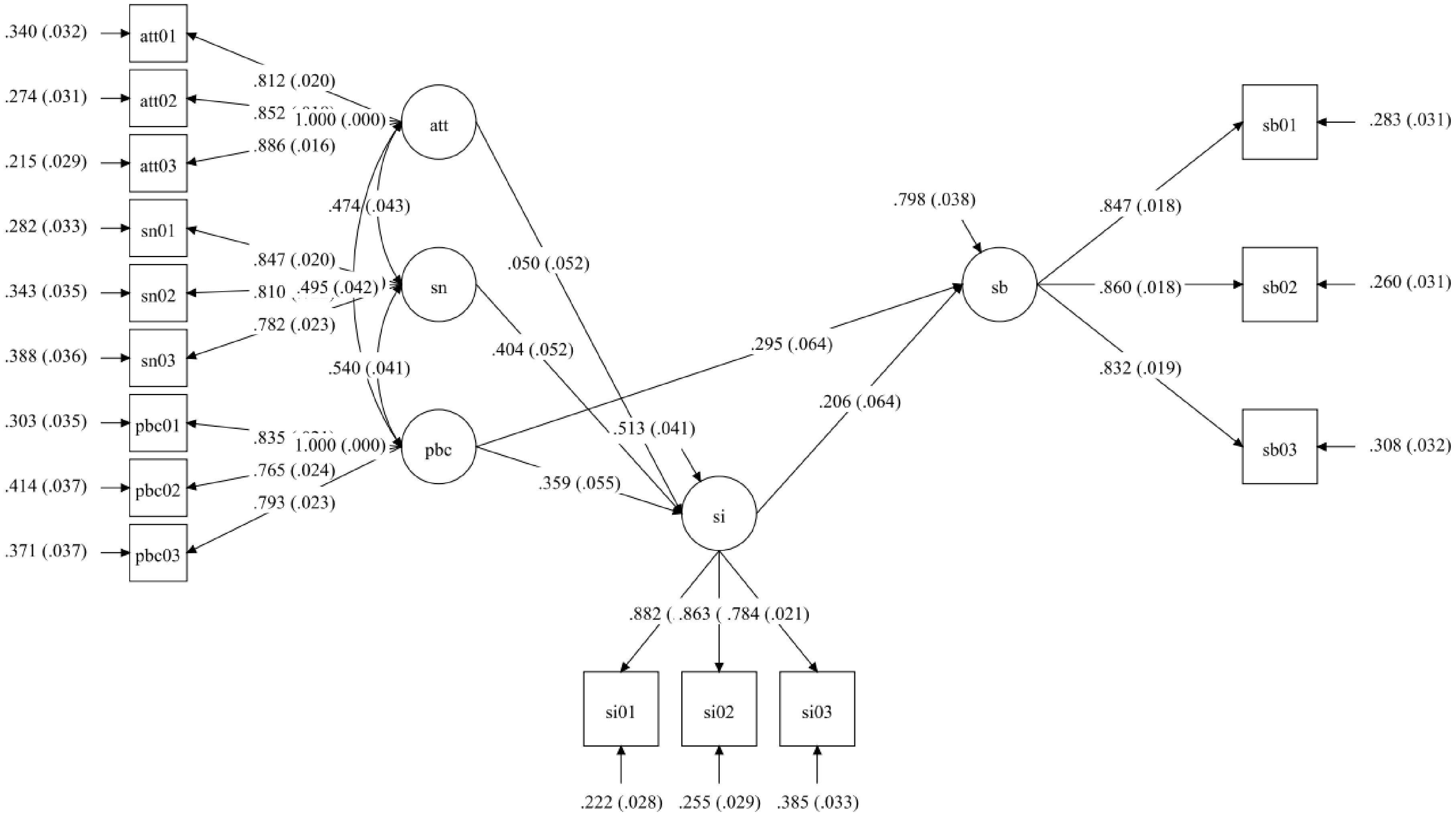

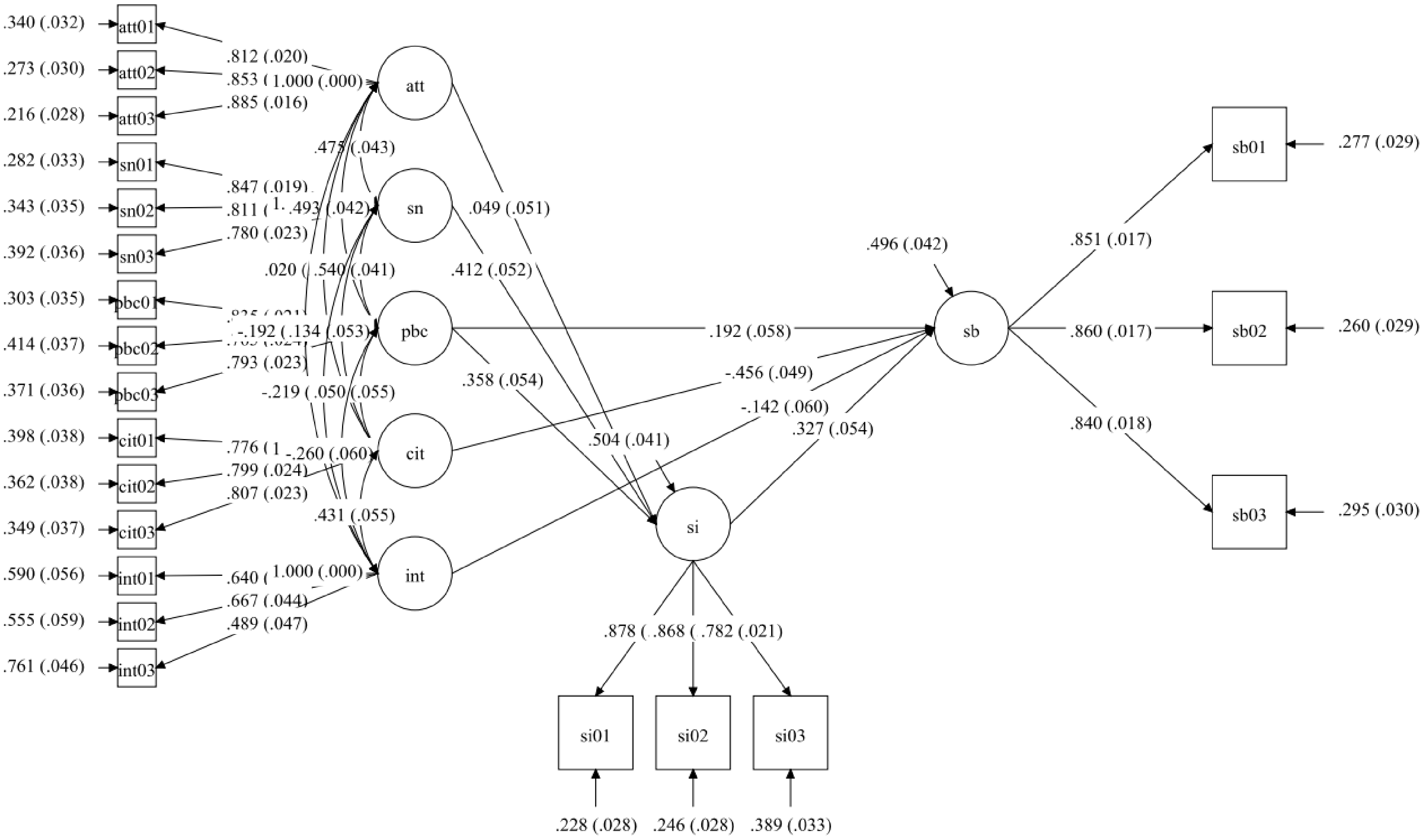

.340 (.032)
att01
.273 (.030)
att02
.216 (.028)
att03
.282 (.033)
sn01
.343 (.035)
sn02
.392 (.036)
sn03
.303 (.035)
pbc01
.414 (.037)
pbc02
.371 (.036)
pbc03
.398 (.038)
cit01
.362 (.038)
cit02
.349 (.037)
cit03
.590 (.056)
int01
.555 (.059)
int02
.761 (.046)
int03
.812 (.020)
1.000 (.000)
.885 (.016)
.847 (.019)
.780 (.023)
.793 (.023)
.799 (.024)
.807 (.023)
.667 (.044)
.489 (.047)
.475 (.043)
.493 (.042)
.540 (.041)
-.192
.134 (.053)
-.219
.050
(.055)
-.260 (.060)
.431 (.055)
att
sn
pbc
cit
int
.049 (.051)
.412 (.052)
.358 (.054)
.504 (.041)
.192 (.058)
-.456 (.049)
-.142 (.060)
.327 (.054)
.496 (.042)
si
sb
.878
.868
.782 (.021)
si01
si02
si03
.228 (.028)
.246 (.028)
.389 (.033)
.851 (.017)
.860 (.017)
.840 (.018)
sb01
sb02
sb03
.277 (.029)
.260 (.029)
.295 (.030)

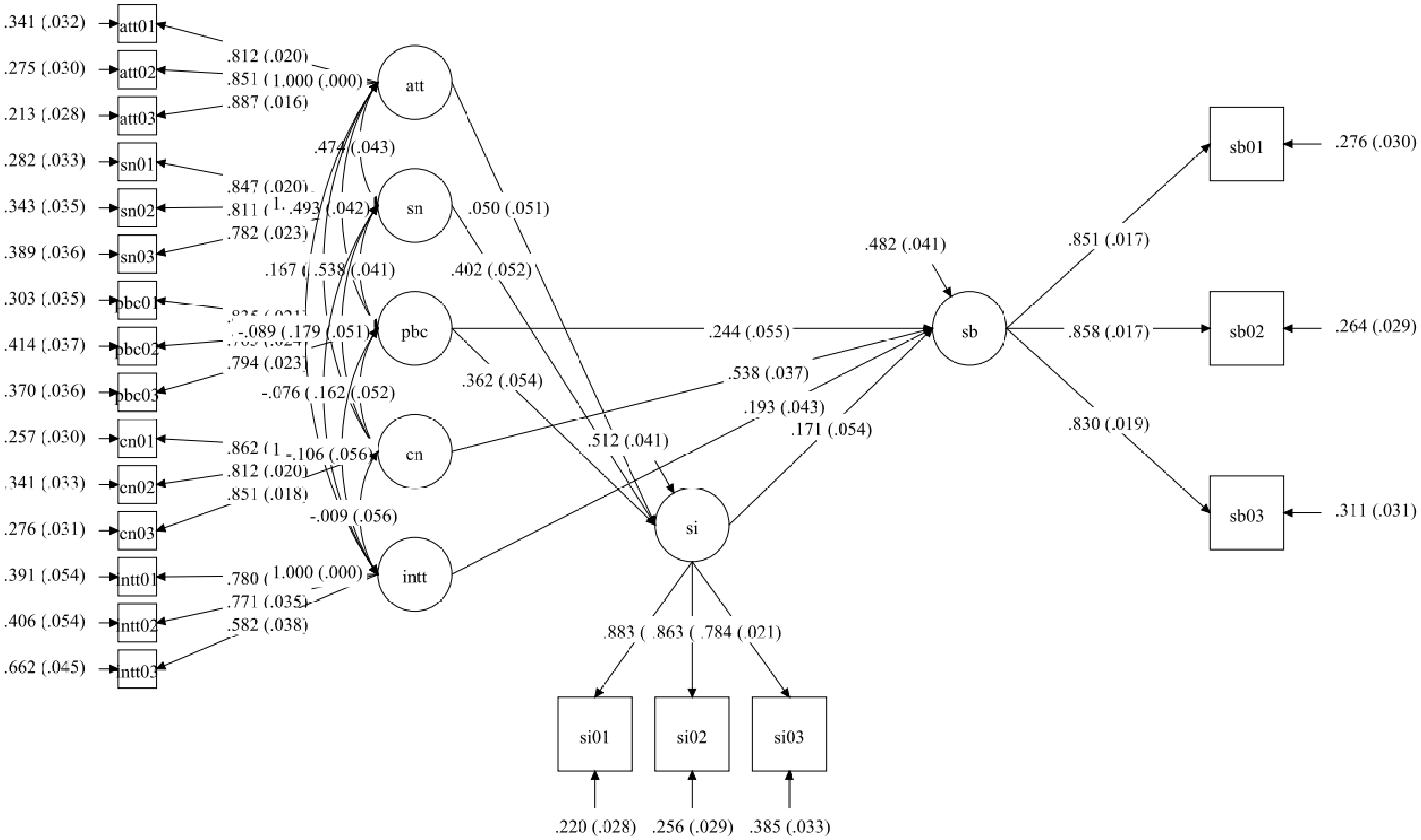

att01
att02
att03
sn01
sn02
sn03
pbc01
pbc02
pbc03
cn01
cn02
cn03
intt01
intt02
intt03
.341 (.032)
.275 (.030)
.213 (.028)
.282 (.033)
.343 (.035)
.389 (.036)
.303 (.035)
.414 (.037)
.370 (.036)
.257 (.030)
.341 (.033)
.276 (.031)
.391 (.054)
.406 (.054)
.662 (.045)
.812 (.020)
1.000 (.000)
.887 (.016)
.474 (.043)
.847 (.020)
.493 (.042)
.782 (.023)
.167
.538 (.041)
.089
.179 (.051)
.794 (.023)
.076
.162 (.052)
.106 (.056)
.812 (.020)
.851 (.018)
-.009 (.056)
1.000 (.000)
.771 (.035)
.582 (.038)
att
sn
pbc
cn
intt
si
sb
.050 (.051)
.402 (.052)
.362 (.054)
.512 (.041)
.244 (.055)
.538 (.037)
.193 (.043)
.171 (.054)
.482 (.041)
.851 (.017)
.858 (.017)
.830 (.019)
sb01
sb02
sb03
.276 (.030)
.264 (.029)
.311 (.031)
.784 (.021)
si01
si02
si03
.220 (.028)
.256 (.029)
.385 (.033)
